# Case Report: Dysfunction of the Paraventricular Hypothalamic Nucleus Area Induces Hypersomnia in Patients

**DOI:** 10.3389/fnins.2022.830474

**Published:** 2022-03-14

**Authors:** Zan Wang, Yu-Heng Zhong, Shan Jiang, Wei-Min Qu, Zhi-Li Huang, Chang-Rui Chen

**Affiliations:** ^1^Department of Neurology, The First Hospital of Jilin University, Changchun, China; ^2^State Key Laboratory of Medical Neurobiology and MOE Frontiers Center for Brain Science, Institutes of Brain Science, Fudan University, Shanghai, China; ^3^Department of Pharmacology, School of Basic Medical Science, Fudan University, Shanghai, China

**Keywords:** lesion, polysomnography, magnetic resonance imaging, case report, hypersomnia, the paraventricular hypothalamic nucleus

## Abstract

**Background:**

Hypersomnia is a common and highly impairing symptom marked by pathological excessive sleepiness, which induces suboptimal functioning and poor quality of life. Hypersomnia can be both a primary (e.g., hypersomnolence disorder) and secondary (e.g., tumors, and head trauma) symptom of disorders. However, its underlying mechanisms remain largely unknown.

**Case Presentation:**

We report that three clinical cases with lesions around the paraventricular nucleus of the hypothalamus (PVH) area showed excessive daytime sleepiness and a prolonged nocturnal sleep lasting more than 20 h per day. Sleep architecture and subjective daytime sleepiness were examined by polysomnography. These cases were presented with stroke, myelin oligodendrocyte glycoprotein (MOG) antibody associated disorders and neuromyelitis optical spectrum disorder (NMOSD), respectively. Magnetic resonance imaging (MRI) showed lesions around the PVH area in all these three patients. After treatment of their primary disorders, their excessive sleep decreased as the PVH area recovered.

**Conclusion:**

Our findings suggest that the PVH may play an essential role in the occurrence of hypersomnia.

## Introduction

Hypersomnia is a complaint of prolonged sleep (excessive daytime sleepiness and a prolonged nocturnal sleep), which results in reduced daily performance and adversely affects the quality of life (Mahowald and Schenck, 2005). As a common sequela of strokes, infections, and tumors (Harris et al., 2014; Barateau et al., 2017), the symptoms of hypersomnia can occur during the course of other neurologic conditions (Kanbayashi et al., 2011). However, imaging studies usually cannot readily elucidate the exact pathogenic mechanism by which hypersomnia develops due to the large-scale brain lesions that can be induced by infections, tumors, and strokes. It is also a relatively uncommon disorder in clinical settings, so the exact mechanism of hypersomnia is not yet understood.

Arousal promotion and maintenance are generally believed to require the support of the multi-wake-promoting system. The ascending reticular activating system (ARAS) is traditionally viewed as an important wake-promoting system (Moruzzi and Magoun, 1949; Carter et al., 2010; Xu et al., 2015). In the last 100 years, more than 16 wake-promoting nuclei have been identified (Moruzzi and Magoun, 1949; Saper et al., 2001; Carter et al., 2010; Xu et al., 2015). Hypersomnia is considered to be closely associated with dysfunction of the wake-promoting system. However, the lesion of a single wake-promoting nucleus, or even three or more wake-promoting nuclei simultaneously, was not found to cause serious hypersomnia (Lu et al., 2006; Fuller et al., 2011; Chen et al., 2016). Further identification of key nuclei and the neural circuitry for hypersomnia is a significant goal for clinicians and researchers.

Here, we reported the three clinical cases with lesions around the paraventricular nucleus of the hypothalamus (PVH) area showed hypersomnia lasting over 20 h per day. They were diagnosed with stroke, myelin oligodendrocyte glycoprotein (MOG) antibody associated disorders (MOG-AD), and neuromyelitis optical spectrum disorder (NMOSD), respectively. After treatment of their primary disorders, their total sleep time (TST) decreased as the lesions in the PVH areas decreased in size. Given that the PVH is not a component of the traditional ARAS system, our findings indicate a new key central node for the maintenance of wakefulness.

## Materials and Methods

### Subjects

Patients were included from the Neurology Department at the First Hospital of Jilin University. This work was approved by the Research Ethics Committee of the First Hospital of Jilin University (2018 trial no. 2018-308). All patients’ relatives were asked to provide written informed consent (2018 trial no. 2018-308).

### Assessments

#### Magnetic Resonance Imaging

All patients completed magnetic resonance imaging (MRI) scanning with a 3.0-T MRI scanner (Siemens Trio Tim 3.0T, Munich, Germany), including axial T1-weighted imaging (T1WI), T2-weighted imaging (T2WI), diffusion-weighted imaging (DWI), fluid-attenuated inversion recovery (FLAIR), and coronal/sagittal scanning, with a slice thickness of 5 mm and a slice spacing of 1 mm.

#### Polysomnography

All patients were monitored for 24 h in the sleep center of our hospital *via* PSG recordings (Compumedics, Australia). Simultaneous monitoring of the following was performed: EEG, electromyogram (EMG), bilateral anterior tibial EMG. All data were analyzed according to the revised interpretation criteria of sleep stages and related events issued by the American Academy of Sleep Medicine (AASM) version 2.1. The core criterion for hypersomnia is excessive subjective sleepiness (total sleep time ≥660 min) (Dauvilliers and Barateau, 2017).

## Results

### Three Patients With Lesions Around the Paraventricular Nucleus of the Hypothalamus Area Showed Hypersomnia

**FIGURE 1:**
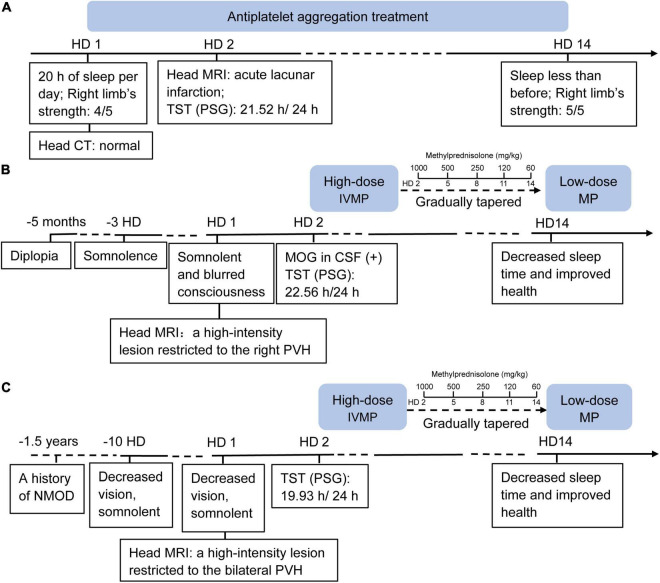
Timeline of case I **(A)**, case II **(B)** and case III **(C)**. HD, hospital day; CT, computed tomography; MRI, magnetic resonance imaging; TST, total sleep time; PSG, polysomnography; IVMP, IV methylprednisolone; MOG, myelin oligo-dendrocyte glycoprotein; CSF, cerebro-spinal fluid; PVH, paraventricular nucleus of the hypothalamus.

**FIGURE 2:**
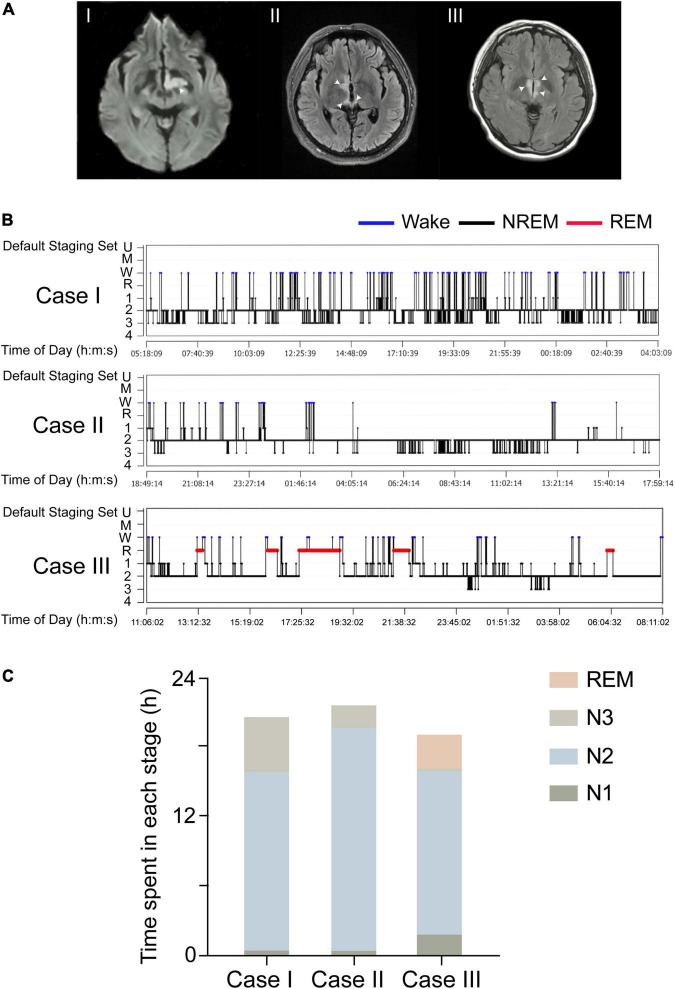
Three patients with lesions in the PVH areas showed hypersomnia. **(A)** DWI signals of the three patients, which suggest strong signals in the left hypothalamus (I). The injury was in the right hypothalamus and showed a slight hypersignal in the FLAIR image (II). The lesion was in the bilateral hypothalamus and showed a slight hyper signal in the FLAIR image (III). White arrows indicate the sites of injury. **(B)** A sleep-structure chart. The N2 stage was dominant, accounting for 74.9% of the total sleep time in case I, 74.1% in case II, and 89.0% in case III. Blue lines represent wakefulness, red lines represent REM sleep, and black lines represent NREM sleep (including stages N1, N2, and N3). **(C)** The sleep duration of N1, N2, N3, and REM in all three cases.

We observed three patients (cases I, II, and III) with central-nervous-system hypersomnia (Figure 1). They had no circadian rhythm disorder before. They showed excessive daytime sleepiness (more than 20 h per day), but they had no signs of cataplexy, sleep paralysis, hypnogogic hallucinations, circadian rhythm disorder, or fever. Cases I, II, III were diagnosed with stroke, MOG-AD, and NMOSDR, respectively. Brain MRI revealed a lesion around the PVH area in each of these three patients: cases I, II, and III displayed DWI with a hypersignal in the left hypothalamus (Figure 2AI and Supplementary Figure 1A), FLAIR sequences with a slight hypersignal in the right hypothalamus (Figure 2AII and Supplementary Figure 1B), and FLAIR sequences with a slight hypersignal in the bilateral hypothalamus (Figure 2AIII and Supplementary Figure 1C), respectively. Sleep structure charts showed that the non-rapid eye movement (non-REM) sleep stage 2 (N2) was dominant, accounting for 74.9% of total sleep time (TST) in case I (Figures 2B,CI), 89.0% in case II (Figures 2B,CII), and 74.1% in case III (Figures 2B,CIII). The clinical characteristics of the three patients before treatment are summarized in Table 1.

**TABLE 1:** Characteristics of the three patients who showed hypersomnia on present.

Characteristic	Case I	Case II	Case III
**Sex**	**Female**	**Male**	**Female**
Age	65	44	19
Days of somnolence	2	3	10
Sleep amount (hours/day)	21.5	22.6	19.9
Body temperature (°C)	36.5	36.5	36.5
Blood pressure (mmHg)	138/85	156/99	124/78
Heart rate (bpm)	67	79	73
Respiration rate (bpm)	18	18	18
Injury site	Left hypothalamus	Right hypothalamus	Bilateral hypothalamus
Total sleep time in a day (min)	1291.0	1354.0	1195.5
NREM stage 1 (min)	27.5	27.0	113.0
NREM stage 2 (min)	966.5	1204.5	886.0
NREM stage 3 (min)	297.0	122.5	11.0
REM (min)	0.0	0	185.5
^#^Sleep efficiency (%)	90.5	97.6	94.8
Diagnosis	Cerebral infarction	Neuromyelitis optical spectrum disorders	Neuromyelitis optical spectrum disorders recrudescence

*^#^Sleep efficiency (SE) is defined as “percentage of time slept of the total time spent in bed.”*

*SE = total sleep time/total recording time × 100%.*

In detail, in case I, a 65-year-old female patient exhibited 20 h of sleep per day, without impairment of consciousness, for more than 2 weeks from the onset of a cerebral infarction because of stroke. Previous sleep rhythm on presentation, she slept through most of the daytime without external stimulation and had weakness in her right limb [medical research council (MRC) grade 4/5]. The patient showed acute lacunar infarction mostly restricted to the left PVH area on MRI images. A 24 h light-dark cycle of PSG showed her TST to be 21.52 h, a sleep efficiency of 90.5%, and no sleep latency. Throughout the entire sleep period, the non-REM sleep stage 2 (N2) was dominant at 16.1 h (74.9%), the N1 sleep time was 0.46 h (2.1%), and the N3 sleep time was 4.95 h (23.0%), and there was no REM sleep detected. PSG data on the respiratory part showed that the apnea-hypopnea index was 2.4 events per hour, and time spent below 90% of saturation of peripheral oxygen (SpO_2_) was 1.75 h (7.4%). Furthermore, considering that the paraventricular hypothalamic nucleus plays an important role in autonomic control, PSG data showed that the periodic leg movements during sleep (PLMS) index was 1 per hour and average heart rate was 92 bpm.

Case II was a 44-year-old man presenting with diplopia for 5 months with a medical history of hypertension and diabetes. On presentation, he was somnolent and experienced blurred consciousness. His eye movement was limited in the upward-downward and the abduction and abduction directions, but his muscle strength was normal. His serum was positive for myelin oligo-dendrocyte glycoprotein (MOG) antibodies (titer: 1:32) and negative for serum anti-aquaporin 4 (AQP4) antibodies. The presence of secondary narcolepsy led to a diagnosis of MOG-AD based on the international consensus criteria (Mahowald and Schenck, 2005). MRI showed a high-intensity lesion restricted to the right PVH area. PSG showed TST to be 22.56 h, his sleep efficiency was 97.6%, and there was no sleep latency. The duration of sleep at the N1, N2, and N3 stages was 0.45 h (2.0%), 20.08 h (89.0%), and 2.04 h (9.0%), and there was no REM sleep or apnea-hypopnea detected. PSG data on the respiratory part showed that the time spent below 90% of SpO_2_ was 3.45 h (16.5%). Furthermore, the PLMS index was 23 per hour and average heart rate was 86 bpm.

Case III was a 19-year-old girl with complaints of decreased vision and 10 days of somnolence. On presentation, her neurological examination was normal, but she had a history of NMOD for 1.5 years and had been treated with immunomodulatory therapy before, so she was diagnosed with NMOD recrudescence. MRI showed a high-intensity lesion restricted to the bilateral PVH. A diagnosis of NMOD recrudescence was made by the detection of a sensitive and highly specific serum autoantibody, AQP4-IgG (Lennon et al., 2004). PSG showed TST to be 19.93 h. Sleep efficiency was 94.8%, and there was no sleep latency. The duration of N1, N2, N3, and REM sleep was 1.88 h (9.5%), 14.77 h (74.1%), 0.18 h (0.9%), and 3.09 h (15.5%), respectively, and no apnea-hypopnea was detected. PSG data on the respiratory part showed that the time spent below 90% of SpO_2_ was 0.16 h (0.9%). In addition, the PLMS index was 44 per hour and average heart rate was 102 bpm.

### Total Sleep Time Decreased With Healing of Lesions Around the Paraventricular Nucleus of the Hypothalamus Area

After 14 days of antiplatelet aggregation treatment (Plavix 75 mg/day), case I slept less than before, and the muscle strength of her right limb was increased. As with cases II and III, after the immunomodulatory therapy with intravenous administration of high-dose (1,000 mg/day) methylprednisolone (IVMP) on three consecutive days, and gradually tapered oral low-dose methylprednisolone (60 mg/day) over 12 days (Figure 1), the two patients both recovered with decreased sleep time and improved health.

**FIGURE 3:**
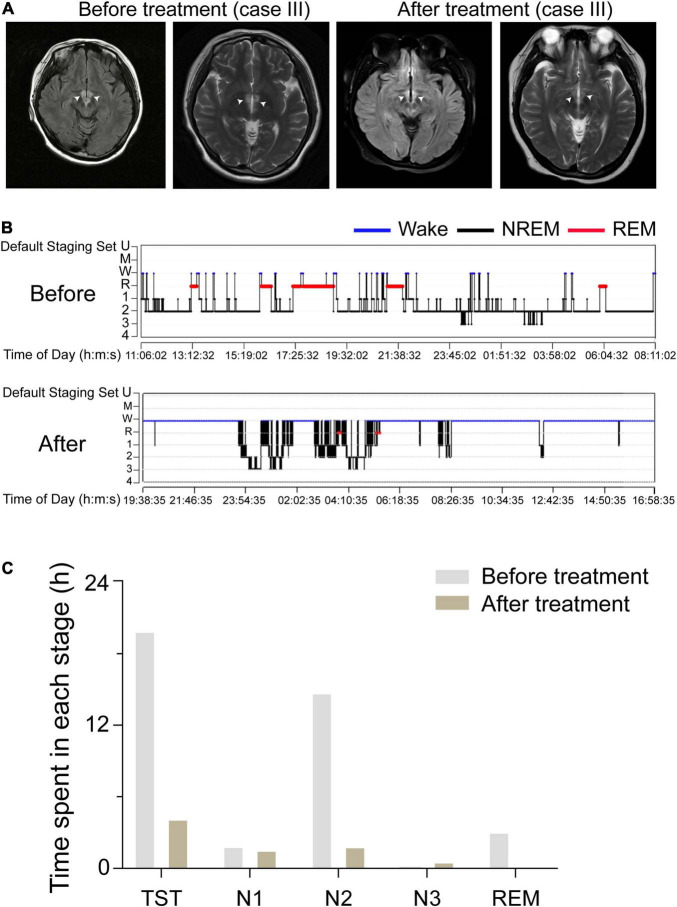
Comparison of the lesion areas and sleep structure of case III before and after treatment. **(A)** The FLAIR of case III, which indicates the lesions were in the bilateral hypothalamus. White arrows indicate the injury sites. **(B)** A sleep-structure chart of case III before and after treatment. Blue lines represent wakefulness, red lines represent REM sleep, and black lines represent NREM sleep (including stages N1, N2, and N3). **(C)** The TST, the duration of N1, N2, and N3 stages, and REM sleep of case III, before and after treatment, during a 24 h sleep–wake cycle.

We compared imaging manifestations and sleep structure charts before and after treatment for case III. We found that her hypothalamic lesions had nearly disappeared by the time of the follow-up MRI, which showed that the lesion was smaller in the FLAIR after treatment (Figure 3A). PSG showed that her TST decreased from 19.93 to 4.18 h per day. The duration of N1, N2, and N3 stages and REM sleep all decreased: N1 sleep went from 113.0 min (9.5%) to 94.5 min (37.7%), N2 sleep from 886.0 min (74.1%) to 112.0 min (44.7%), N3 sleep from 35.5 min (14.2%) to 11 min (0.9%), and REM sleep from 185.5 min (15.5%) to 0.0 min (0.0%); see Figures 3B,C. These results suggest that dysfunction of the PVH area might act as a central node for the occurrence of hypersomnia.

## Discussion

We describe the lesions restricted to the unilateral/bilateral PVH area induced hypersomnia in three patients. They showed excessive daytime sleepiness (more than 20 h per day) and were monitored for 24 h in the sleep center. PSG recordings from these patients showed that N2 sleep was strikingly dominant, indicating that these patients slept stably and were not easily awakened. We found that the TST decreased with healing of the lesions around the PVH in one of these patients, indicating that this patient was concomitantly better able to stay awake.

Hypersomnia is one of the most common symptoms of many neurological disorders and mental diseases, including major depressive disorder, bipolar disorder, Alzheimer’s disease (AD), and Parkinson’s disease (PD) (Purba et al., 1994; Manaye et al., 2005; Baloyannis et al., 2015). In line with our findings, a robust reduction in the number of PVH neurons has been found in patients with these diseases (Purba et al., 1994; Manaye et al., 2005; Baloyannis et al., 2015). Thus, our present findings provide evidence that dysfunctions of the PVH may contribute to the pathogenesis of hypersomnia in these diseases.

In the last 100 years, more than 16 wake-promoting nuclei have been identified. Studies on lethargic encephalitis by von Economo (1930) have identified that hypothalamic damage is crucial for sleep-related symptoms, including hypersomnia. However, among these wake-promoting nuclei, only the lateral hypothalamic area (LH) orexinergic and parabrachial complex (PB) glutamatergic neurons have been shown to be related to hypersomnia (Gerashchenko et al., 2003; Adamantidis et al., 2007; Kaur et al., 2013; Yokota et al., 2015). Patients with PD, AD, Kleine-Levin Syndrome, and idiopathic hypersomnia, in which LH orexinergic and PB glutamatergic neurons are thought to function normally, still show hypersomnolence (Bollu et al., 2018). Furthermore, dysfunctions of the PVH induced hypersomnia-like behaviors in mice (Chen et al., 2021) and patients with narcolepsy type 1 showed reduced numbers of corticotropin-releasing hormone neurons in the PVH (Shan et al., 2022), further supporting the importance of the PVH in arousal controlling and maintenance. Therefore, our case report first provides clinical evidence that dysfunctions of the PVH area led to hypersomnia in patients.

There have been reports on the possible pathology associated with hypersomnia. Kume et al. (2015) reported a temporal lobe lesion caused NMOSD-related hypersomnia because of secondary damage to orexin neurons in the hypothalamus. Another study demonstrated that hypersomnia occurred after paramedian thalamic stroke due to the interruption of noradrenergic and dopaminergic activating transmission ascending from the brainstem reticular formation to the thalamus (Bassetti et al., 1996). However, the pathogenesis for hypersomnia has not been fully understood and its optimal treatment strategies need to be established.

It has been many years that researchers hypothesized that loss of hypocretin/orexin signaling in narcolepsy, a type of hypersomnia, was caused by autoimmune reactions (Bassetti et al., 2019). Other studies have found that the presence of autoreactive CD8^+^ and CD4^+^ T cells in narcolepsy, providing further support to the pivotal role of specific T cells in causing neuronal damage in human narcolepsy (Adamantidis et al., 2007; Yokota et al., 2015). Besides, some studies reported the association between AQP4-IgG, MOG antibodies and hypersomnia (Kaur et al., 2013; Bollu et al., 2018; Chen et al., 2021) or disorder associated with MOG antibodies and hypersomnia (Liu et al., 2020), with regression of hypersomnia after appropriate treatment of the autoimmune disease. On the other hand, it has been recently shown that an up-regulation of anti-MOG antibodies can be modulated by prolactin (Shan et al., 2022), a hormone correlated with the onset of hypersomnia (Kume et al., 2015). These findings indicated the potential role of the inflammatory/autoimmune pathogenesis underlying hypersomnia in these three cases with lesions of PVH area.

Admittedly, there are some limitations in our report. As for the sleep evaluation, we did not perform morning-evening questionnaire or the multiple sleep latency test. Meanwhile, we did not detect serum melatonin to evaluate circadian rhythm. As for the diagnosis, we did not examine the hypocretin of cerebrospinal fluid in these patients and did not use 7T MRI to scan the hypothalamus. Apart from limits in methodology, the clinical component of our present study was also limited by the availability of data from cases I and II after their treatment because these patients declined to undergo post-treatment PSG monitoring; however, these two patients reported that their post-treatment sleep durations had returned to normal levels. We observed that the reduction of time spent awake as measured *via* PSG monitoring showed an earlier recovery than that revealed *via* MRI, suggesting that PSG monitoring may be a more direct and sensitive tool for predicting the prognosis of PVH injury.

In conclusion, dysfunction of the PVH areas led to hypersomnia in three patients with different protopathic diseases, and our findings identified a possible brain target for understanding the pathology and etiology of hypersomnia. Furthermore, considering a possible inflammatory/autoimmune pathogenesis in the presence of hypersomnia, it is important to evaluate inflammatory/autoimmune pathogenesis (especially in the case of MOG-AD and NMOSDR) in the presence of an acute/subacute onset of hypersomnia, especially in consideration of the regression of the symptom following treatment.

## Data Availability Statement

The original contributions presented in the study are included in the article/Supplementary Material, further inquiries can be directed to the corresponding authors.

## Ethics Statement

The studies involving human participants were reviewed and approved by the Research Ethics Committee of the First Hospital of Jilin University. The patients/participants provided their written informed consent to participate in this study.

## Author Contributions

ZW collected the clinical data. Y-HZ and SJ analyzed the data. ZW, SJ, and C-RC wrote the manuscript. All authors helped with the revision of the manuscript.

## Conflict of Interest

The authors declare that the research was conducted in the absence of any commercial or financial relationships that could be construed as a potential conflict of interest.

## Publisher’s Note

All claims expressed in this article are solely those of the authors and do not necessarily represent those of their affiliated organizations, or those of the publisher, the editors and the reviewers. Any product that may be evaluated in this article, or claim that may be made by its manufacturer, is not guaranteed or endorsed by the publisher.

## Supplementary Material

Supplementary Figure 1 Magnetic resonance imaging (MRI) images of three cases before treatment, related to Figure 2. **(A)** Sagittal scanning MRI with different layers of case I. **(B)** Sagittal scanning (left) and coronal scanning (right) MRI of case II. **(C)** Sagittal scanning MRI with different layers of case III. Red arrows indicate the sites of injury.
