# Supplementary figures and images for: Case Report: Dysfunction of the Paraventricular Hypothalamic Nucleus Area Induces Hypersomnia in Patients
Source: Front Neurosci. 2022 Mar 14;16:830474. doi: 10.3389/fnins.2022.830474 (PMC8964012; doi:10.3389/fnins.2022.830474)

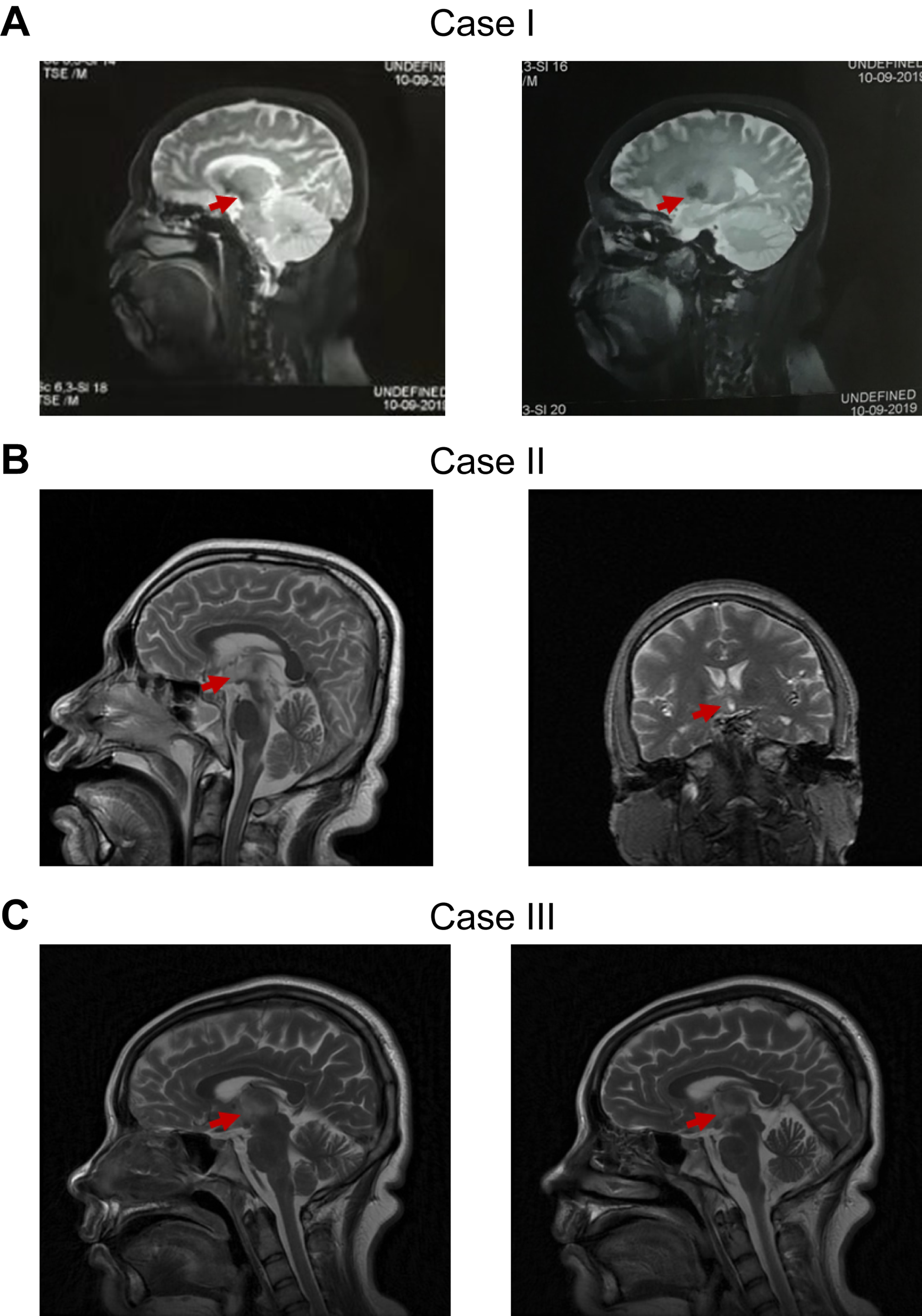

Supplement: Supplementary Figure 1 — Magnetic resonance imaging (MRI) images of three cases before treatment, related to Figure 2. (A) Sagittal scanning MRI with different layers of case I. (B) Sagittal scanning (left) and coronal scanning (right) MRI of case II. (C) Sagittal scanning MRI with different layers of case III. Red arrows indicate the sites of injury. [file Image_1.TIF]
